# Child Loss and Cognitive Health Among Older Adults

**DOI:** 10.1093/geroni/igaf122.507

**Published:** 2025-12-31

**Authors:** Sneha Mani, Muqi Guo, Lindsay Kobayashi, Alden Gross

**Affiliations:** Johns Hopkins Bloomberg School of Public Health, Baltimore, Maryland, United States; University of Michigan School of Public Health, Ann Arbor, Michigan, United States; University of Michigan, Ann Arbor, Michigan, United States; Johns Hopkins Bloomberg School of Public Health, Baltimore, Maryland, United States

## Abstract

The loss of a child, a severe life adversity, is associated with long-term health risks for parents, including increased mortality, cardiovascular diseases, and mental health issues. However, its potential impact on later-life cognitive health remains understudied. We examine the association between child loss and cognitive function using data from 3,456 participants aged ≥60 from the Longitudinal Aging Study in India and 1,927 participants aged ≥55 from the Mexican Health and Aging Study. Child loss was defined by self-reported loss of at least one child aged ≥5 years or (18% in India, 10% in Mexico) or ≥ 25 years (11% in India, 6% in Mexico). Cognitive function was measured using country-specific co-calibrated cognitive factor scores using measures from the Harmonized Cognitive Assessment Protocol (HCAP). A multivariable-adjusted, country-stratified linear regression model was used to examine the relationship between child loss and cognitive function. Compared to individuals who never experienced child loss, those who lost a child aged ≥5 years and those who lost a child aged ≥25 years exhibited -0.07 SD (95% CI: -0.12 to -0.01) and -0.09 SD (95% CI: -0.16 to -0.02) lower cognitive scores in India, and -0.13 SD (95% CI: -0.24 to -0.01) and -0.06 SD (95% CI: -0.20 to 0.08) lower scores in Mexico, respectively. These findings suggest that child loss across the life course may represent a significant risk factor for poorer later-life cognitive function in older adults.

